# Covering patient’s perspective in case‐based critical review articles to improve shared decision making in complex cases

**DOI:** 10.1111/hex.13108

**Published:** 2020-07-23

**Authors:** Louis‐Rachid Salmi, Pierre Côté, Christine Cedraschi

**Affiliations:** ^1^ Univ. Bordeaux, ISPED Centre INSERM U1219 Bordeaux Population Health Bordeaux France; ^2^ INSERM ISPED Centre INSERM U1219 Bordeaux Population Health Bordeaux France; ^3^ CHU de Bordeaux Pole de Sante Publique Service d’information Medicale Bordeaux France; ^4^ Center for Disability Prevention and Rehabilitation Canadian Memorial Chiropractic College Univ. of Ontario Institute of Technology Oshawa ON Canada; ^5^ Ontario Technical University Oshawa ON Canada; ^6^ Division of General Medical Rehabilitation Geneva Faculty of Medicine Geneva Switzerland; ^7^ Division of Clinical Pharmacology & Toxicology Multidisciplinary Pain Centre Univ. Hospitals Geneva University Geneva Switzerland

**Keywords:** evidence‐based medicine, evidence‐based practice, low back pain, patient’s perspective, practice guidelines

## Abstract

**Background:**

The patient has always been at the centre of the evidence‐based medicine model. Case‐based critical reviews, such as best‐evidence topics, however, are incomplete reflections of the evidence‐based medicine philosophy, because they fail to consider the patient's perspective. We propose a new framework, called the ‘Shared Decision Evidence Summary’ (ShaDES), where the patient's perspective on available treatment options is explicitly included.

**Methods:**

Our framework is grounded in the critical appraisal of a clinical scenario, and the development of a clinical question, including patient characteristics, compared options and outcomes to be improved. Answers to the clinical question are informed by the literature, the evaluation of its quality and its potential usefulness to the clinical scenario. Finally, the evidence synthesis is presented to the patient to facilitate the formulation of an evidence‐informed decision about the treatment options.

**Key results:**

Using three similar but contrasted clinical scenarios of patients with low back pain, we illustrate how considering the patient's preferences on the proposed treatment options impact the bottom line, a synthetic formulation of the answer to the focused question. ShaDES includes clinical and psychosocial components, transformed in a searchable question, with a full search strategy.

**Conclusions:**

ShaDES is a practical framework that may facilitate clinical decisions adapted to psychological, social and other relevant non‐clinical characteristics of patients.

## 1 INTRODUCTION

Evidence‐Based Medicine (EBM) is the ‘conscientious, explicit and judicious use of current best evidence in making decisions about the care of individual patients’.^1^ Proponents of EBM have always advocated that ‘information comes from two principal sources, the individual patient and research’.^2^ The EBM approach has later included the clinical expertise of the practitioner^1^ and the accessibility of health‐care resources.^3^

The patient has always been at the centre of EBM. Some early EBM publications included case scenarios.^4^ Typically, an EBM approach starts with developing a clinical question, which can reflect a treatment, diagnostic or prognosis issue, and answering it by searching for, reviewing and critically appraising the relevant literature. The answer to the clinical question is applied to the patient^5^ and allows for a shared decision making. The approach has been formalized by structuring some case‐based critical reviews as critically appraised topics (CATs)^6^, ^7^, ^8^ or best‐evidence topic (BET) reviews.^9^, ^10^, ^11^

Failure to incorporate the patient’s perspective into guidelines has been criticized as a major obstacle to successfully implementing EBM with individual patients.^12^ These criticisms are reflected in the development of patient‐centred outcomes^13^, ^14^ and shared decision making.^13^, ^15^ The inclusion of patient choice is one of the cornerstones of theories linking EBM to quality of individual care and performance of care organization.^16^ We believe that, similarly, case‐based critical reviews are incomplete reflections of the EBM philosophy. Although these reviews start from and end with the patient,^5^ BETs and other case‐based critical reviews usually fail to include shared decision making, considering the patient’s perspective on expected outcomes that can result from the available treatment options identified in the literature. Notably, the current format of BETs and other case‐based critical reviews appears less useful when several treatment options are available to the practitioner and the patient. In such complex instances, according to Barry,^13^ patients are too often left feeling in the dark about how their problem is managed and how to navigate in the array available to them. As all patient‐centred approaches, these reviews should respect patient’s values, preferences and expressed needs, key dimensions of quality of care.^13^

In this paper, we review the structure of current BETs and similar case‐based reviews and introduce an alternative approach, called ‘Shared Decision Evidence Synthesis’ (ShaDES). ShaDES improves the currently used approaches by integrating the relevant scientific evidence and the perspective of a patient to choose an acceptable evidence‐based treatment. We illustrate the application of ShaDES using three clinical scenarios.

## 2 STRUCTURE OF BETS AND OTHER CASE‐BASED CRITICAL REVIEWS

**FIGURE 1:**
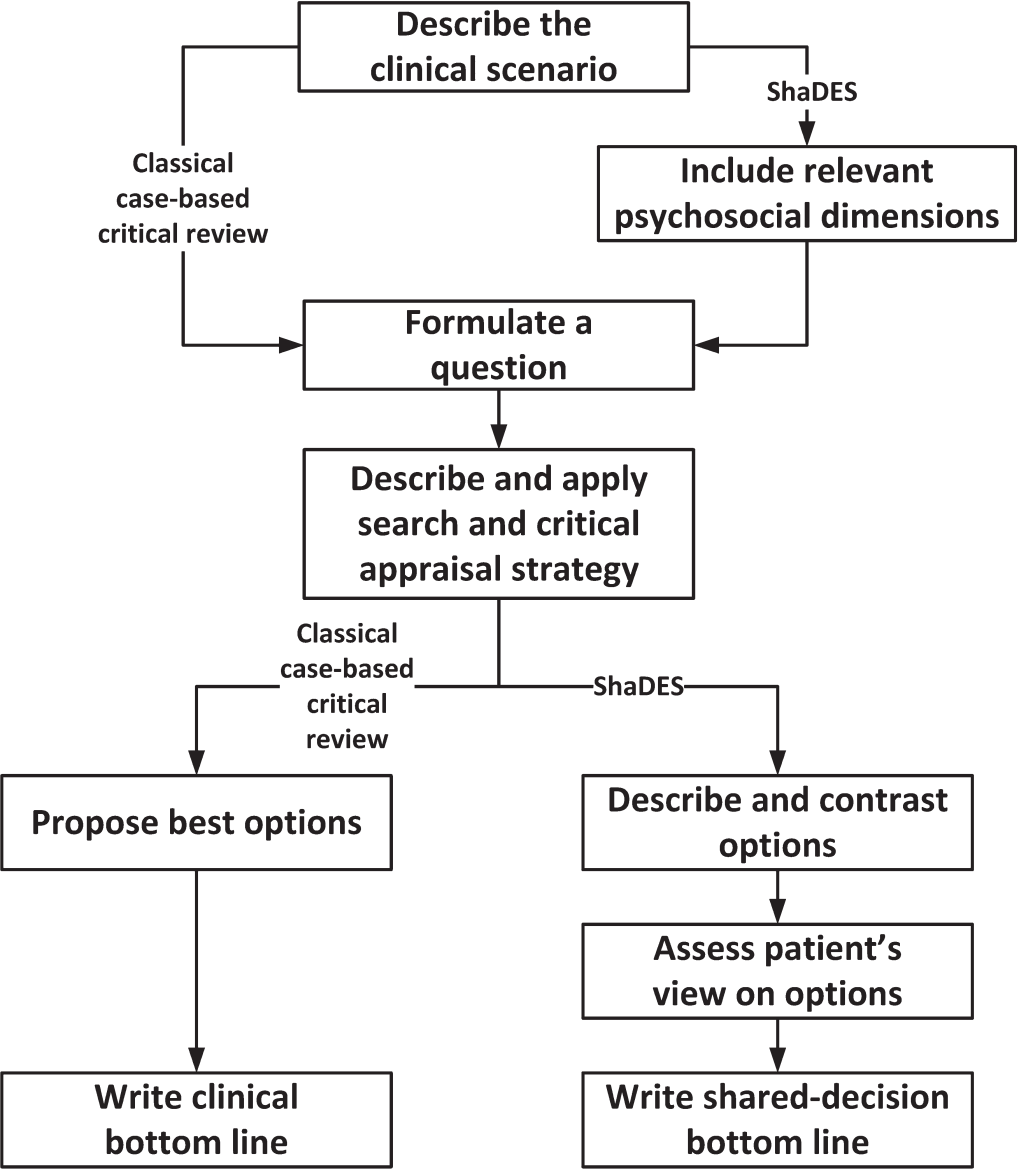
An algorithm integrating steps of current case‐based critical reviews and the proposed Shared Decision Evidence Synthesis

The typical CAT or BET starts with a clinical scenario, which describes a patient’s complaint, medical history, clinical examination and diagnostic tests, and prognostic or treatment challenges (Figure 1). The scenario is then transformed into a clinical question, which includes five distinct components: ‘In [type of patients/population], is [intervention considered, diagnostic test, or risk or prognostic marker] [modifier according to the question: better, not inferior, effective, accurate, reliable] to [type of question: treat, identify and predict] [name of disorder or outcome to be improved or predicted]’. The most structured method to frame the clinical question is the PICOT system,^5^ where P stands for Population, I for Intervention, C for Comparison, O for Outcome, and T for Time to outcome. For example, for a case reported by Dy et al^17^ of a 78‐year‐old woman with cholangiocarcinoma and presenting with jaundice and worsening abdominal pain, the focused clinical question could be ‘In patients with advanced cancer, would biliary stenting, compared with standard palliative care, have a positive impact on survival, disease progression, symptoms and complications in the short term?’.

Answers to the clinical question are then searched from the literature, by retrieving evidence‐based guidelines, systematic reviews or original studies.^5^, ^18^ This process involves identifying clinically relevant keywords and combining them in search equations, describing the retrieved studies, critically appraising the studies and extracting key results.^19^ Results of the process, regarding the available evidence, its strengths and weaknesses and its potential usefulness to answer the clinical question are finally translated into what is called the ‘clinical bottom line’. The clinical bottom line is a synthetic formulation of the answer to the focused question initially raised. For instance, in the above illustration,^17^ the bottom line could be ‘For this patient, there is no evidence that the benefits of biliary stenting would outweigh the risk related to this procedure’.

## 3 CLINICAL SCENARIOS NEEDING DIFFERENT BOTTOM LINES

The typical case‐based critical review is adapted to situations where the bottom line proposes a straightforward answer to the clinical question. The bottom line, derived from the evidence in the literature, allows the practitioner to decide whether to prescribe a treatment, or how to interpret results of a test. We believe the current format of BETs and similar case‐based critical reviews is not well adapted to situations where evidence is weak or lacking, or when there are several options among which the practitioner, and therefore the patient, must choose. We present three clinical scenarios of patients with similar clinical history and examination, but different psychosocial (anxiety, social isolation, apprehension and worries of pain) and socioeconomic context (country, occupation and possibly health insurance). We demonstrate how different patient profiles may result in different evidence‐based therapeutic options and management of the patients.

### 3.1 Common background

The three patients complain of low back pain (LBP) without specific lesions. They use analgesic and were prescribed exercises by a physiotherapist. These interventions provided moderate benefits. The patients consult their general practitioner (GP) for the next available options. All patients are currently working or willing to work, but they express worries about their ability to continue.

### 3.2 Clinical vignette #1

Sophie is a 40‐year‐old bus driver in her small French home town and is also responsible for planning work assignments for a team of bus drivers. Her new manager is difficult and not inclined to consult others for their opinions about work organization. Sophie is married to Georges, a bank employee; they have two teenage children. When Sophie comes home from work, she has to supervise the children’s homework and prepare the meal. Georges contributes little to home chores, which sometimes leads to conflicts.

Sophie has chronic recurrent LBP. Her most recent episode started six weeks ago while gardening. She felt a sharp pain in her lower back. She takes analgesics and has remained at work. However, her home activities are limited and her husband is worried that she cannot complete her ‘chores’. Sophie feels guilty and fears that her manager believes that she might not be able to do her job appropriately. She believes that there is something wrong with her back and that her pain is caused by pinched nerve due to her occupation. Sophie was prescribed exercises by her physiotherapist and, although she never missed her appointments, she is fearful that doing exercises will harm her back and make her condition worse. She is distressed and depressed.

### 3.3 Clinical vignette #2

Mary is a 45‐year‐old registered nurse employed in a large Canadian hospital. She recently applied for a head nurse position and is waiting for the decision. Mary is married to Mark, an accountant, and they have three children aged 8, 10, and 12 years. Mary spends most of her spare time building a sailing boat with the help of her spouse and children.

She has suffered from recurrent LBP for eight months. Mary uses muscle relaxants and she enthusiastically participated in active physical therapy. These interventions provided moderate benefits. Her pain is particularly intense at the end of the day, and she occasionally experiences sharp bouts of pain at work, which makes her anxious. She knows that chronic LBP is a benign disorder and that fear of pain can be disabling. She has never been on sick leave because of her back, but she now fears that she may not be able to continue, which amplifies her anxiety. She is very concerned that she will not be promoted at work. This makes her even more anxious, because she has been working hard for years to obtain the promotion and has gained the support of her colleagues. She is considering stopping building the boat despite the help of her family.

### 3.4 Clinical vignette #3

Gloria has been suffering from LBP for the past four years. Her pain started insidiously and has been persistent for the past two years. She is 46‐year‐old, married, with an 18‐year‐old daughter. She was employed as a secretary in a small factory in Switzerland. She also worked as a part‐time cook, and launched a successful blog which publishes her best recipes. However, she has to rest most of the time because of the pain. Her condition has not improved despite various courses of NSAIDs and duloxetine and physical therapy pool‐ and land‐based exercises. She describes her family as supportive but she feels extremely guilty, because she cannot perform the activities she used to do when she was ‘healthy’. Overwhelmed by pain, she is pessimistic and gloomy, invaded by dark thoughts and feelings. She tries to stay active in her everyday life, but she does not succeed. When asked about what gives meaning to her life, she can hardly respond.

### 3.5 Commentary

Current evidence‐based recommendations for the clinical management of chronic LBP are clear and include many options.^20^, ^21^, ^22^, ^23^, ^24^ In a traditional BET/CAT model, the clinical question would be the same for the three patients: In patients with chronic LBP, how effective and safe are available therapeutic options in reducing pain and improving function? The therapeutic options which are supported by evidence of varying quality and could be considered by practitioners include manual therapy, yoga and cognitive‐behavioural therapy (CBT). However, Sophie, Mark and Gloria have different psychological profiles, and their preference must be considered when selecting the treatment. Indeed, Sophie and Mary are still active with their occupation and family life despite their pain and anxiety, and they expect to be able to carry on with their activities. However, Gloria has stopped all her activities, even the ones that she could engage in. Interacting with her family is a source of support for Mary, while not always be helpful for Sophie and are a source of depressive feelings for Gloria. While Sophie and Mary consider that treatment may be helpful, Gloria’s negative appraisal also applies to treatments.

## 4 SHARED DECISION EVIDENCE SYNTHESIS

ShaDES includes four steps (Figure 1): First, the clinician builds the clinical and psychological scenario. Second, the clinical information collected in the history and examination informs the literature search and critical appraisal of the retrieved evidence. Third, the clinician synthesizes the evidence, as is done when developing decision aids.^25^, ^26^, ^27^, ^28^, ^29^ Finally, the clinician and patient enter in a shared decision process where the patient expresses his/her preferences regarding the available treatment options.

### 4.1 Starting with a clinical and psychological scenario

Implementing ShaDES starts with understanding the clinical scenario by describing the patient, his/her medical and personal history and clinical examination, and identifying diagnostic, prognostic or treatment issues (Figure 1). As seen in our three vignettes, the collection of relevant clinical information is complete when all factors that could impact the prognosis and the perspective of the patient have been collected. The scenario is then transformed into a structured question that includes five components: ‘In patients with recurrent LBP and unsatisfactory results of evidence‐based medication and exercise (P), what are the effects of manual therapy, yoga and CBT (I & C) on pain and function (O)?’

### 4.2 Elements common with BETs and other case‐based critical reviews

This part is not specific to ShaDES, and readers can refer to Elwyn et al^15^ and Straus et al^5^ on principles of EBM and shared decision making. Readers can also find an example of search strategy and selection criteria for non‐specific low back pain in Balagué et al.^23^ The search strategy is provided with keywords and equations combining these keywords, along with a description of the main studies eventually found and critically appraised. Similarly, one can refer to Oliveira et al^20^ and Ikemoto et al^22^ to see typical presentations of synthesis results, or any above‐cited case‐based critical reviews^6^, ^7^, ^9^, ^10^, ^11^ for focused presentations of outputs of reviews.

### 4.3 Integrating patient preferences

In the next step of ShaDES, the evidence synthesis is presented to the patient to facilitate the formulation of an evidence‐informed decision about the treatment options. To successfully achieve this step, one can use, for instance, O’Connor et al.’s approach to construct decision aids.^25^, ^26^, ^27^, ^28^, ^29^ Basically, each available option is presented to the patient with an explanation of: (1) what the treatment is; (2) what are the benefits; and (3) what are the risks and side effects. (eg https://decisionaid.ohri.ca/AZsumm.php?ID=1045 for the decision aid for Spinal Manipulation for Low Back Pain; consulted June 2nd, 2020). In such decision aids, the patient is invited to express his/her feeling by using sliders or cursors through visualization of the relevant dimensions (outcome possibly improved, risks, side effects…) that are important to the patient.

In ShaDES, we propose to present the evidence in a comparative table (Table 1). The table can be completed by incorporating patient’s feelings, expressed by asking questions about the patient’s view on credibility and individual fit of the option (Do you think [name of the option] could work for you?), its effectiveness and effects (Is what [name of the option] can do to [improve/reduce] [name of the outcome] important to you?) and its disadvantages (Are you concerned with [name of the risk or side effect]?). For example, feelings of Sophie, Mary and Gloria (Table 1) could be expressed by asking, after explaining what is yoga, ‘Do you thing yoga could work for you?’ ‘Is what yoga can do to reduce pain important for you’ etc.

**TABLE 1:** Available options to treat patients with chronic low back pain who did not benefit from an evidence‐based intervention that included medication and exercise

Dimension	Manual therapy	Yoga	Cognitive‐behavioural therapy
What is it?	Application of loads by using levers and thrusts to a spinal joint beyond its range of motion (manipulation) and passive movements within the range of motion (mobilization)	Mind‐body practice to help patients with health problems manage their condition and reduce their symptoms	Structured mental health counselling to make aware of inaccurate or negative thinking
How effective is it? (one line by outcome)	Pain: small benefit	Pain: small benefit	Pain: small benefit
	Function: small benefit	Function: small to moderate benefit	Function: small benefit
What are the risks associated with it? (one line by risk or side effect)	Pain (1 out of 20) Neurological complaints (rare)	Increase in back pain (1 out of 10); similar to exercise No serious adverse events	Temporary stress
What are the patient’s feeling regarding the type of intervention?	Sophie: Believes that a pinched nerve can be fixed by manipulation or mobilization	Sophie: Has heard from friends that yoga can ‘fix’ many kinds of problems	Sophie: Expresses that anything that could help her cope with her anxiety would be good
	Mary: Considers that it could be a good complement to ease compliance to exercises	Mary: Does not believe it can be effective	Mary: Expresses that it might help her deal with her stress
	Gloria: Expresses she is ready to give a try to anything that could help her and that she has not tested yet	Gloria: Finds it hard to believe that it could help her	Gloria: Considers that it could help her identify meaningful objectives
What are the patient’s expectations regarding the benefits?	Sophie: Assumes that manipulation or mobilization might trigger a stressful or traumatic perception in the back	Sophie: Hopes this treatment will decrease pain and will allow her avoid taking medication	Sophie: Thinks that this treatment may help her in many ways, including in dealing with her fear of exercising
	Mary: Considers manual therapy as a first choice treatment for LBP	Mary: Does not anticipate much in terms of results, but ready to give it a try	Mary: Expects this treatment to help her better identify her goals, considering her priorities and also her fears
	Gloria: Has tested various physical treatments and hopes she will find a therapist with a ‘magic touch’	Gloria: Does not anticipate much in terms of results, and not convinced she should give it a try	Gloria: Hopes this treatment might help her find goals and meaning to her life
What are the patient’s concerns about the risks?	Sophie: Is afraid manual therapy might increase pain	Sophie: Feels somehow uneasy and worried to hear that yoga is labelled an alternative treatment	Sophie: Fears that getting a psychological treatment may lead her family and boss to consider that her pain is not real and ‘is in her head and not in her back’
	Mary: Is bothered because she does not know whether side effects are immediate or gradual and what she should pay attention to	Mary: Fears that stretching movements may cause pain and harm	Mary: Is concerned that she might feel irrational describing her personal and intimate worries
	Gloria: Fears manual therapy may not prove more effective than the other therapies and that she might be disillusioned	Gloria: Fears it might increase her pain and make life impossible for her	Gloria: Is afraid she might end up discovering that her life is really worthless and meaningless
Summary of the evidence	Manual therapy is an effective intervention for LBP, but patients’ fears about the treatment need to be addressed.	Yoga is an effective intervention for chronic LBP, but patients’ fears about the treatment need to be addressed.	CBT is an effective treatment options for patients who do not want physical treatment; it can also be used in combination with manual therapy and yoga.
Application to the patients (bottom line)	Preferred option for Mary, but not Gloria	An option for Sophie, but not Mary, and still less for Gloria	Preferred option for Sophie preferred treatment for Gloria, along with treatment of her depression

The last part of ShaDES is, as in existing CATs and BETs, the bottom line. To improve the usability of ShaDES, we recommend summarizing the pros and cons of each treatment to assist patients with understanding the trade‐offs necessary to achieve patient‐centred outcomes. For example, the last line of Table 1 summarizes why CBT is likely to be chosen by Sophie and Gloria, and manual therapy by Mary.

### 4.4 Application

If we now consider how the elements of our proposed model can be applied to the clinical situations described above, the following issues and questions need be emphasized and addressed:

Sophie: Sophie was not reassured by her GP, because of her fear of pain or recurrent injury. The clinical and psychological question for Sophie is whether her challenges with her recurrent LBP and the unsatisfactory results of evidence‐based medication and exercise (P) could be better addressed with cognitive‐behavioural therapy and yoga (I & C) directed at pain and function (O). For such conditions, there is strong evidence that CBT is more effective for pain, functional status and behavioural outcomes than placebo/no treatment/waiting list controls. CBT requires the identification of meaningful and realistic goals to allow Sophie to address her catastrophizing with pain and activity and thus improve her readiness to engage in active pain self‐management. Sophie may also consider yoga as she expresses positive expectations towards such a treatment, but limited evidence exists to support its effectiveness compared with standard treatments.^20^, ^22^

Mary: The GP could reassure Mary by addressing her concerns regarding her work. In this instance, the clinical and psychological question is whether her challenges with her recurrent LBP and the unsatisfactory results of evidence‐based medication and exercise (P) could be better addressed with a multimodal programme including spinal manipulation and cognitive‐behavioural therapy (I & C); these interventions would target pain and functional limitation and her expectations regarding what she wants to achieve in both her occupational and personal life (O). Options considered by Mary include manual therapy and CBT. The aims of CBT are to help Mary identify meaningful and realistic goals and learn to cope with pain and stress. Because Mary expressed strong positive beliefs regarding physical treatment, the GP prescribes a short course of spinal manipulation/mobilization.^20^

Gloria: The GP felt unable to address adequately Gloria’s pain problems associated to feeling of hopelessness and worthlessness, which also included feelings of self‐depreciation, shame and guild, along with passive suicidal thoughts. Here, the clinical and psychological question would focus on her difficulties with her recurrent LBP and unsatisfactory results of evidence‐based medication and exercise (P) in relation with her depressive disorder. In this instance, a referral to a psychiatrist or psychologist would be indicated to adequately address her feelings and her maladaptive cognitions and behaviours related to movement (I & C), which may contribute to improve pain and function (O). The symptoms here go beyond psychological distress or psychosocial dysfunction and refer instead to a depressive disorder, and referral to a psychiatrist or psychologist in the presence of such orange flags should be considered.^30^, ^31^ Further, using education strategies might help addressing Gloria’s negative affect and beliefs, and assisting her in the identifications of meaningful goals for her to achieve.

## 5 DISCUSSION

To our knowledge, ShaDES is the first framework that explicitly includes patients’ perspectives in case‐based critical reviews. Our illustration represents real life, where practitioner and patients are usually confronted to more complex situations than are usually depicted in guidelines or clinical scenarios.^12^ Thus, ShaDES is closer to the full model of EBM than other short reviews, such as BETs. ShaDES may also more ethical, because one cannot make informed choices without information, even if scientific information is lacking, scarce, poor or equivocal in the literature.^4^, ^32^, ^33^, ^34^, ^35^

We focused on including the patient’s perspective. Nevertheless, we still have to consider the practitioner’s experience, or availability of resources,^1^, ^3^ or even, as suggested by Siminoff et al., the patient’s family preferences.^36^ The inclusion of physician’s experience has become possible with electronic medical records^37^, ^38^ and automatic search of a practitioner’s databases or a hospital data warehouses.^39^ Issues of availability and access to resources, such as delays to access professionals in the specific context where patients are seen (manual, yoga and CBT therapists and psychiatrists in our example) would also have to be addressed. For instance, our three vignettes occurred in different countries where access to chiropractic care varies. In Switzerland, chiropractors are primary care practitioners and reimbursed by the health‐care system. In Canada, chiropractors have been recognized since 1945 and their services are reimbursed by various insurers through the universal health‐care system. In France, chiropractic was recognized in 2002 but is not paid by the universal health‐care system. Family preferences could be relevant, for example, when they have to take the patient to a remote location to access to a specific care.

Since the onset of evidence‐based practice, clinicians have struggled to incorporate preferences in shared decision making. Kelly et al^40^ underscore the need to determine the optimal methods for shared decision tools and to facilitate their incorporation in daily clinical practice. These authors also provide other tools, such as inpatient portals, that could replace or complement O’Connor et al’ approach in ShaDES. How the patient’s perspective and preferences are integrated in shared decision making still requires further considerations. Indeed, the patient’s perspective may include preferences for non‐clinical outcomes (eg doing house chores or spending time on a hobby) and the ability to actively cope with what is required to achieve these outcomes. This requires a consideration of biological, physical or psychological factors, values, motivations and beliefs, as well as comorbidities. Therefore, given a specific patient’s clinical scenario, a clinician may face an evidence review that yields many possible decisions. We have proposed three scenarios with similar clinical and demographic backgrounds, but different outcomes, to illustrate the importance of psychological factors and patient’s values and beliefs in the management of low back pain. Circumstances and evidence alignment, however, could vary in time and between patients. It has been shown, for instance, that dimensions considered important vary with patient’s characteristics such as age and gender;^41^ other relevant characteristics to consider might be type of disorder (cancer…) or circumstances (busy clinics…). One also should consider that the patient’s perspective might not always yield a clear recommended course of action, but rather a clear set of choices among options identified in available guidelines or a specific literature search. Also, patients’ values and preferences might sometime lead to options of scientifically questionable value; for instance, acupuncture was considered an acceptable albeit weak option in previous recommendations^23^ and yoga, now considered an acceptable option,^24^ might be considered by some patients or practitioners as questionable. Further research should be conducted on risks associated with presenting patients with such options, and how risk communication techniques can improve shared decision making.^42^ However, even when patients’ perspectives and preferences were incorporated in the development of clinical practice guidelines, the clinician should adapt the decision to the needs of the patient.^12^ Finally, decision aids are effective in facilitating shared decision making in actual clinical circumstances,^43^ but the way to include patients preferences in the process of developing a BET needs further research. For instance, the ability of tools to measure patient preferences to capture the outcome desired by patients^14^ should be validated in studies respecting metrology standards^44^ and specific recommendations regarding patient‐centred outcomes.^14^

Further research needs to focus on the integration of key characteristics of the clinician’s psychology, such as empathy,^45^ affective attitude^46^ or ability to provide cognitive reassurance,^47^ known to lower anxiety and distress and to be associated with better clinical outcomes.^45^ We also need methods to improve the development of effective decision aids or other tools for shared decision making^15^, ^43^ and to integrate them in daily clinical practice, including in busy practices where available time might be an issue. In such instances, we believe our approach is pragmatic, as it is based on questions to assess patient preferences that are close to the usual patient‐practitioner encounter; these questions are also a good reflection of the ‘Choice Talk, Option Talk, Decision Talk’ model of Elwyn et al.^15^ We need research on the effect of implementing ShaDES in clinical practice, including comparisons with shared decision approaches, such as qualitative studies investigating patient adherence and satisfaction, and quantitative research on effectiveness.^16^ We also need to better train caregivers to focus critical reading on clinically relevant issues such as proximity of population covered, relevance of outcome measurement, and magnitude and precision of effect sizes. Above all, if we are to discuss the need to incorporate the patients’ perspectives in all decision making, training must address the development of caregivers to explain evidence to patients. Basic training of health‐care professionals also must convey the importance of having the same rigour when documenting indication of a procedure and baseline and outcome measure in records and information systems.

## 6 CONCLUSION

We have proposed a new way to present case‐based critical reviews, which might better reflect the original philosophy of EBM. Our proposition puts a strong emphasis not only on clinical but also on psychosocial components, aimed to point to a question that can be developed into a search strategy. This proposition thus offers a practical framework that may contribute to clinical decisions using relevant psychological, social and other characteristics of the patient and of his/her environment. The applicability and impact of such an approach, which specifically considers values, preferences and needs of patients, remain to be tested.

## CONFLICT OF INTEREST

The authors declare they have no conflict of interest.

## Data Availability

Data sharing is not applicable to this article as no new data were created or analysed in this study.

## References

[1] Sackett DL , Rosenberg WMC , Gray JAM , Haynes RB , Richardson WS . Evidence based medicine: what it is and what it isn't. BMJ. 1996;312(7023):71‐72. 8555924 10.1136/bmj.312.7023.71 PMC2349778

[2] Medicine Evidence‐Based Working Group . Evidence‐based medicine. A new approach to teaching the practice of medicine. JAMA. 1992;268(17):2420‐2425. 1404801 10.1001/jama.1992.03490170092032

[3] Charles C , Gafni A , Freeman E . The evidence‐based medicine model of clinical practice: scientific teaching or belief‐based preaching? J Eval Clin Pract. 2011;17(4):597‐605. 21087367 10.1111/j.1365-2753.2010.01562.x

[4] Oxman AD , Sackett DL . Guyatt GH, the evidence‐based Medicine Working Group. Users' guides to the medical literature. I. How to get started. JAMA. 1993;270(17):2093‐2095. 8411577

[5] Straus S , Richardson WS , Glasziou P , Haynes RB . Evidence‐Based Medicine. How to Practice and Teach EBM. Edinburgh: Elsevier Churchill Livingstone; 2005.

[6] Bowden SC , Harrison EJ , Loring DW . Evaluating research for clinical significance: Using critically appraised topics to enhance evidence‐based neuropsychology. Clin Neuropsychol. 2014;28(4):653‐668. 23463942 10.1080/13854046.2013.776636

[7] Crowell MS , Tragord BS , Al T . Deyle G . Integration of critically appraised topics into evidence‐based physical therapist practice. J Orthop Sports Phys Therap. 2012;42(10):870‐879. 22814199 10.2519/jospt.2012.4265

[8] Callander J , Anstey AV , Ingram JR , Limpens J , Flohr C , Spuls PI . How to write a Critically Appraised Topic: evidence to underpin routine clinical practice. Br J Dermatol. 2017;177(4):1007‐1013. 28967117 10.1111/bjd.15873

[9] Mabvuure NT , Klimach S , Eisner M , Rodrigues JN . An audit of best evidence topic reviews in the International Journal of Surgery. Int J Surg. 2015;17:54‐59. 25819136 10.1016/j.ijsu.2015.03.014

[10] Slee H . Can throat examination distinguish between bacterial and viral infective agents? Emerg Med J. 2010;27(10):790‐792. 20852285 10.1136/emj.2010.101329

[11] Vymazal T . Massive hemorrhage management‐a best evidence topic report. Ther Clin Risk Manag. 2015;11:1107‐1111. 26251606 10.2147/TCRM.S88878 PMC4524472

[12] McCartney M , Treadwell J , Maskrey N , Lehman R . Making evidence based medicine work for individual patients. BMJ. 2016;353:2452. 10.1136/bmj.i2452 27185764

[13] Barry MJ , Edgman‐Levitan S . Shared decision making–pinnacle of patient‐centered care. N Engl J Med. 2012;366(9):780‐781. 22375967 10.1056/NEJMp1109283

[14] Greenhalgh J , Long AF , Brettle AJ , Grant MJ . Reviewing and selecting outcome measures for use in routine practice. J Eval Clin Pract. 1998;4(4):339‐350. 9927249 10.1111/j.1365-2753.1998.tb00097.x

[15] Elwyn G , Frosch D , Thomson R , et al. Shared decision making: a model for clinical practice. J Gen Intern Med. 2012;27(10):1361‐1367. 22618581 10.1007/s11606-012-2077-6 PMC3445676

[16] Greenhalgh J , Dalkin S , Gibbons E , et al. How do aggregated patient‐reported outcome measures data stimulate health care improvement? A realist synthesis. J Health Serv Res Policy. 2018;23(1):57‐65. 29260592 10.1177/1355819617740925 PMC5768260

[17] Dy SM , Harman SM , Braun UK , Howie LJ , Harris PF , Jayes RL . To stent or not to stent: an evidence‐based approach to palliative procedures at the end of life. J Pain Symptom Manage. 2012;43(4):795‐801. 22464354 10.1016/j.jpainsymman.2011.12.269 PMC4696003

[18] DiCenso A , Bayley L , Haynes RB . Accessing pre‐appraised evidence: fine‐tuning the 5S model into a 6S model. Evid‐Based Nursing. 2009;12(4):99‐101. 10.1136/ebn.12.4.99-b 19779069

[19] White CM , Sanders Schmidler GD , Butler M , et al. AHRQ Methods for Effective Health Care Understanding Health‐Systems' Use of and Need for Evidence To Inform Decisionmaking. Rockville, MD: Agency for Healthcare Research and Quality (US); 2017. 29611913

[20] Oliveira CB , Maher CG , Pinto RZ , et al. Clinical practice guidelines for the management of non‐specific low back pain in primary care: an updated overview. Eur Spine J. 2018;27(11):2791‐2803. 29971708 10.1007/s00586-018-5673-2

[21] Maher C , Underwood M , Buchbinder R . Non‐specific low back pain. Lancet. 2017;389(10070):736‐747. 27745712 10.1016/S0140-6736(16)30970-9

[22] Ikemoto T , Miki K , Matsubara T , Wakao N . Psychological treatment strategy for chronic low back pain. Spine Surg Relat Res. 2019;3(3):199‐206. 31440677 10.22603/ssrr.2018-0050 PMC6698517

[23] Balagué F , Mannion AF , Pellisé F , Cedraschi C . Non‐specific low back pain. Lancet. 2012;379(9814):482‐491. 21982256 10.1016/S0140-6736(11)60610-7

[24] Wieland LS , Skoetz N , Pilkington K , Vempati R , D'Adamo CR , Berman BM . Yoga treatment for chronic non‐specific low back pain. Cochrane Database Syst Rev. 2017;1:Cd010671. 28076926 10.1002/14651858.CD010671.pub2 PMC5294833

[25] Elwyn G , O'Connor A , Stacey D , et al. Developing a quality criteria framework for patient decision aids: online international Delphi consensus process. BMJ. 2006;333(7565):417‐421. 16908462 10.1136/bmj.38926.629329.AE PMC1553508

[26] O’Connor AM , Bennett CL , Stacey D , et al. Decision aids for people facing health treatment or screening decisions. Cochrane Database Syst Rev. 2009;CD001431. 19588325 10.1002/14651858.CD001431.pub2

[27] O'Connor AM , Légaré F , Stacey D . Risk communication in practice: the contribution of decision aids. BMJ. 2003;327(7417):736‐740. 14512487 10.1136/bmj.327.7417.736 PMC200814

[28] Stacey D , Legare F , Col NF , et al. Decision aids for people facing health treatment or screening decisions. Cochrane Database Syst Rev. 2014;Cd001431. 24470076 10.1002/14651858.CD001431.pub4

[29] Stacey D , Legare F , Lewis K , et al. Decision aids for people facing health treatment or screening decisions. Cochrane Database Syst Rev. 2017;4:Cd001431. 28402085 10.1002/14651858.CD001431.pub5 PMC6478132

[30] Main CJ , Sullivan MJ , Watson PJ . Risk identification and screening In: Main CJ, Sullivan MJ, Watson PJ, eds. Pain Management: Practical Applications of the Biopsychosocial Perspective in Clinical and Occupational Settings. Edimburgh: Churchill Livingstone Elsevier; 2008:97‐134.

[31] Nicholas MK , Linton SJ , Watson PJ , Main CJ . Decade of the Flags" Working G. Early identification and management of psychological risk factors ("yellow flags") in patients with low back pain: a reappraisal. Phys Therap. 2011;91(5):737‐753. 21451099 10.2522/ptj.20100224

[32] Bell NR , Dickinson JA , Grad R , Singh H , Kasperavicius D , Thombs BD . Understanding and communicating risk: Measures of outcome and the magnitude of benefits and harms. Can Fam Physician. 2018;64(3):181‐185. 29540382 PMC5851390

[33] Christine PJ , Kaldjian LC . Communicating evidence in shared decision making. Virtual Mentor. 2013;15(1):9‐17. 23356800 10.1001/virtualmentor.2013.15.1.ecas1-1301

[34] Naci H , Ioannidis JPA . Evaluation of wellness determinants and interventions by citizen scientists. JAMA. 2015;314(2):121‐122. 26068643 10.1001/jama.2015.6160

[35] Thomas G . Reverse evidence based medicine. Pan Afr Med J. 2013;16:89. 24711879 10.11604/pamj.2013.16.89.2782 PMC3976658

[36] Siminoff LA . Incorporating patient and family preferences into evidence‐based medicine. BMC Med Inf Decision Making. 2013;13(S3):S6. 10.1186/1472-6947-13-S3-S6 PMC4029304 24565268

[37] Frankovich J , Longhurst CA , Sutherland SM . Evidence‐based medicine in the EMR era. N Engl J Med. 2011;365(19):1758‐1759. 22047518 10.1056/NEJMp1108726

[38] McGinn T . Putting meaning into meaningful use: A roadmap to successful integration of evidence at the point of care. JMIR Med Inform. 2016;4(2):e16. 27199223 10.2196/medinform.4553 PMC4891572

[39] Dietrich G , Krebs J , Fette G , et al. Ad hoc information extraction for clinical data warehouses. Meth Inform Med. 2018;57(1):e22‐e29. 29801178 10.3414/ME17-02-0010 PMC6193399

[40] Kelly MM , Hoonakker PLT , Coller RJ . Inpatients sign on: An opportunity to engage hospitalized patients and caregivers using inpatient portals. Med Care. 2019;57(2):98‐100. 30520834 10.1097/MLR.0000000000001043 PMC6331235

[41] Mazzi MA , Rimondini M , van der Zee E , Boerma W , Zimmermann C , Bensing J . Which patient and doctor behaviours make a medical consultation more effective from a patient point of view. Results from a European multicentre study in 31 countries. Patient Educ Couns. 2018;101(10):1795‐1803. 29891103 10.1016/j.pec.2018.05.019

[42] Barnes AJ , Hanoch Y , Miron‐Shatz T , Ozanne EM . Tailoring risk communication to improve comprehension: Do patient preferences help or hurt? Health Psychol. 2016;35(9):1007‐1016. 27183307 10.1037/hea0000367

[43] Légaré F , Stacey D , Turcotte S , et al. Interventions for improving the adoption of shared decision making by healthcare professionals. Cochrane Database Syst Rev. 2014;15(9):CD006732. 10.1002/14651858.CD006732.pub2 20464744

[44] Streiner DL , Norman GR . Health Measurement Scales. A Practical Guide to their Development and Use, 3rd edn Oxford: Oxford University Press; 2003.

[45] Derksen F , Bensing J , Lagro‐Janssen A . Effectiveness of empathy in general practice: a systematic review. Br J Gen Pract. 2013;63(606):e76‐e84. 23336477 10.3399/bjgp13X660814 PMC3529296

[46] Mazzi MA , Rimondini M , Deveugele M , et al. What do people appreciate in physicians' communication? An international study with focus groups using videotaped medical consultations. Health Expect. 2015;18(5):1215‐1226. 23796047 10.1111/hex.12097 PMC5060825

[47] Holt N , Pincus T , Vogel S . Reassurance during low back pain consultations with GPs: a qualitative study. Br J Gen Pract. 2015;65(639):e692‐e701. 26412846 10.3399/bjgp15X686953 PMC4582882

